# Has Virtual Care Arrived? A Survey of Rural Canadian Providers During the Early Stages of the COVID-19 Pandemic

**DOI:** 10.1177/11786329221096033

**Published:** 2022-05-17

**Authors:** Lindsay Burton, Kathy L Rush, Mindy A Smith, Matthias Görges, Leanne M Currie, Selena Davis, Mona Mattei, Jennifer Ellis

**Affiliations:** 1 School of Nursing, University of British Columbia-Okanagan, Kelowna, BC, Canada; 2 Patient Voices Network, Vancouver, BC, Canada; 3 Department of Family Medicine, Michigan State University, East Lansing, MI, USA; 4 Department of Anesthesiology Pharmacology & Therapeutics, University of British Columbia, and Research Institute, BC Children’s Hospital, Vancouver, BC, Canada; 5 School of Nursing, University of British Columbia, Vancouver, BC, Canada; 6 Department of Family Practice, University of British Columbia, Vancouver, BC, Canada; 7 Kootenay Boundary Division of Family Practice, Trail, BC, Canada

**Keywords:** COVID-19, virtual care, telehealth, rural

## Abstract

We investigated the uptake and perceptions of virtual care solutions by rural Canadian primary and specialist providers during the early phase (May-June 2020) of the COVID-19 pandemic. A web-based, cross-sectional survey of rural primary and specialty care providers examined types of virtual care platforms used (eg, phone, video), appointment length, experience and satisfaction with the solution used, plans for future use of virtual care, and patients’ use of virtual care services. Targeted participants were actively-practicing providers in rural Western Canada who were emailed an invitation for the study and its survey link. Fifty-nine providers (26% response rate) completed the survey. During the pandemic, 78% of providers reported using virtual care for more than 60% of their appointments, while only 3% did so frequently pre-pandemic. Most providers used phone consultations, despite believing that video provided a better virtual visit. Key barriers included workflow interruptions, unique concerns about quality of care, remuneration and sustainability, or poor internet access and bandwidth for both providers and patients. The key opportunity noted was improved access to care. While most virtual care visits were not conducted using video technologies, overall virtual care resulted in high provider satisfaction, while not increasing workload. Virtual care will continue to play an important role in future rural care practice; however, sustainability will require both provider-level and system-level changes.

## Introduction

People living in rural and remote Canadian communities have traditionally faced significant barriers to accessing both primary and specialized healthcare. However, the COVID-19 pandemic, and rapid pivot to virtual visits to promote patient and provider safety, led to improved healthcare access for many.^1^ Benefits have included decreased emergency room visits, preserving healthcare resources, and lessening the spread of COVID-19.^2^ Despite its benefits, virtual care, or “visits that take place between patients and providers via communications technology in real time from any location,”^3^ presents challenges and uncertainties especially for rural providers. In a recent scoping review of virtual primary care implementation, De Vera et al^4^ reported that more than half of the included papers focused on inequities, specifically lack of rural access to internet, smartphones, and internet bandwidth. Only one Canadian non-empirical-based paper on virtual primary care was included, despite Canada being the world’s second-largest country with a highly rural geography.^5,6^ Little is known about rural Canadian primary care provider’s implementation and use of virtual care during the pandemic, but understanding their perspectives considering the existing inequities in access to virtual care is critical to better support its future use. Further, prior survey approaches were limited to provider’s opportunity to open-ended questions on their perceptions and experiences of virtual care.

The purpose of this study was to determine rural provider’s virtual care perceptions and use of virtual care during the pandemic. Research questions included: (a) what has been the provider’s use of virtual care (which virtual care platforms were used, and how this choice affected appointment length), (b) what has been the provider’s overall experience and satisfaction with virtual care, and (c) what are the provider’s plans for future virtual care use?

### Review of literature

Pre-pandemic, well documented barriers to health care access for rural communities included a shortage of primary care providers; limited local specialized services; travel time to healthcare facilities, complicated by seasonal and geographic variations; and economic hardship (eg, travel and accommodations are often at patients’ personal expense).^7-9^ While virtual care has been touted as equalizing access regardless of geographical location, evidence suggests that the COVID-19 pandemic may have widened disparities for rural communities. For example, in a study using population/administrative data from Ontario, Canada, researchers reported the proportion of total virtual visits provided to patients residing in rural areas (vs urban areas) in Ontario declined significantly between 2012 and 2020.^10^

Both to urban-based and rural providers have experienced challenges to virtual care usage, including independently selecting from diverse virtual care tools, scheduling difficulties, and complicated billing.^11,12^ A qualitative review of COVID-19 pandemic virtual care highlighted infrastructure, institutional, and human factors which can greatly impact uptake.^12^ However, compared to urban providers, rural providers face greater digital infrastructure inequities such as inadequate bandwidth and inconsistent internet connectivity.^13,14^Sixty percent of rural households in Canada lack high speed internet access ^15^; a glaring disparity compared to the 3% of urban households who face the same barrier.^16^ Consistent with these differences, Canadian researchers found that providers in rural areas of Southwestern Ontario were more likely to consider inconsistent Wi-Fi and limited connectivity as barriers to incorporating virtual visits within the practice setting than urban providers.^17^

Several Canadian and US studies have surveyed primary providers usage and experiences with virtual care during the pandemic.^18,19^ While the research overall demonstrates an increase in virtual care across studies, there has been considerable variability in reporting pre-post pandemic comparisons,^18,19^ with limited disaggregation of rural-urban usage differences. Saiyed et al^19^ found that only 65% of US physicians (primary/specialty) felt that the physician-patient relationship was unimpaired using virtual care, with those reporting good video and audio quality being 3.7 times more likely to enjoy and be satisfied with virtual care; yet, rural and urban comparisons were not provided. Additional concerns, noted in a review of virtual care, were the scarcity of information about communication technology use when interacting with specialists and sustainable funding concerns for rural providers adopting virtual care.^20^

Rural concerns and challenges around virtual care will require solutions.^21^ Rural providers’ views of current and future use of virtual care are an important driver for ongoing planning to ensure long-term, equitable access and care quality. Further, to maximize their support, it is important to identify their challenges, the nature and frequency of system use, satisfaction with available virtual care solutions, and the supports and strategies needed to promote sustained use.

## Methods

### Design

This cross-sectional study used a web-based survey to obtain a portrait of rural providers use of virtual care during the early COVID-19 pandemic.^22,23^ This study followed the Strengthening the Reporting of Observational Studies in Epidemiology (STROBE) Statement for reporting cross-sectional studies.

### Context/setting

This study took place in a mountainous rural region of Western Canada. Providers in the region deliver care through 26 primary care clinics, 1 urgent primary care clinic, 4 maternity clinics, and 7 community health centers/hospitals. At the time of the study: May-June 2020, all providers were advised to use virtual care—telephone, web-based, and other means of telecommunications technology—whenever possible to assess, triage, and provide advice.^24^ While in-person visits began to expand in May 2020, the majority of visits were conducted virtually throughout the data collection period.^25^

### Sample

Following research ethics board approval (REB # H20-01328) in partnership with a local community-based non-profit organization of family physicians, we targeted healthcare providers including: local primary care physicians, nurse practitioners (NP), midwives, and physician/NP specialists. We recruited participants using our partner organization’s database of approximately 230 providers actively working in the region. Providers were emailed an invitation for the study and a survey link by a team member of our partner organization.

### Data collection/measures

A researcher-generated survey was sent to providers in May 2020, and was followed up with one reminder in June 2020, using the online survey platform Checkbox (Checkbox Survey Inc, Watertown, MA, USA). The online survey began with a consent cover page which explained the study purpose, approximate time commitment, and compensation, with continuation to the survey implying consent. Providers, who completed the survey, were paid a half-hour sessional fee commensurate with their professional designation as established by the primary care working group. The survey (Supplemental Appendix A) was developed in partnership with the local provider group and research team members. The survey contained 6 sections that asked a series of closed (categorical and Likert scale) and open-ended questions about: (1) provider characteristics (eg, gender, age, provider type); (2) virtual care platform usage (types such as phone or video, preference for use depending on appointment, reasons for choosing platform, current percentage of virtual appointments, use prior to COVID-19 pandemic) and appointment length (eg, how many appointments/day, length of virtual appointment); (3) virtual care satisfaction (eg, how virtual care has changed satisfaction, satisfaction with patient care, appropriate appointments for virtual care, benefits of virtual care, barriers) and experience (eg, ease of use for providers and patients, problems with virtual visits, and workload considerations); (4) future virtual care plans (eg, virtual care use after COVID-19 pandemic, virtual care to enhance future care, fee code to incentive virtual care use, any additional supports needed); (5) practices’ patient characteristics (panel size, subsets of patients forgoing care, patients seeking care more frequently); and (6) provider awareness of their patients’ use of other virtual care services (are patients using other virtual platforms outside of practice).

### Analysis

Cross-sectional survey data were analyzed descriptively. No power analysis was performed as there were no pre-stated hypotheses. Responses with less than 50% completion rate were excluded from analyses (n = 6). Chi-square tests were performed to investigate differences in virtual care primary modality (telephone only vs telephone and video vs Zoom) and categories of satisfaction, ease of virtual care, and virtual care appointment lengths by provider type (ie, generalist vs specialist). Kruskal-Wallis rank sum and Mann-Whitney *U* tests were conducted to determine, if there were significant differences in satisfaction, ease of virtual care, and virtual care appointment lengths between provider type and virtual care modality. Correlation analysis was run for satisfaction, ease of virtual care, and virtual care appointment lengths. The sample did not meet the assumptions for skewness or kurtosis for multiple regression model analyses. Data analyses were conducted using R version 3.6.1 (R Foundation for Statistical Computing, Vienna, Austria). Open-ended survey questions were open-coded independently by 2 researchers to reach inter-rater reliability of 80% or higher using NVivo version 12 (QSR International Pty Ltd., Melbourne, Australia). Once initial coding was completed, similar codes were clustered into derived themes using research team consensus. Qualitative results were merged with quantitative results using a weaving approach to exemplify findings.^26^

## Results

Fifty-nine providers completed the survey, a response rate of 25.6% (see Table 1).

**Table 1.** Summary of participant demographics and virtual-care experience.

	Overall (N = 59) (%)	Generalist (N = 36) (%)	Specialist (N = 21) (%)	*P*
Gender				
Female	35 (60)	20 (56)	15 (71)	.42
Male	21 (36)	15 (42)	6 (29)	
Non-binary	1 (2)	^a^	^a^	
Age (y)				
25-34	4 (7)	^a^	^a^	.43
35-44	26 (44)	17 (47)	9 (43)	
45-54	17 (29)	8 (22)	9 (43)	
55-64	8 (14)	^a^	^a^	
65 or older	2 (3)	^a^	^a^	
Virtual care modality				
Phone	32 (54)	21 (58)	9 (43)	.37
Phone and video	24 (41)	14 (39)	10 (48)	
Zoom	3 (5)	1 (3)	2 (9)	
Satisfaction with virtual care				
Satisfied	38 (64)	26 (72)	10 (48)	.17
Neutral	14 (24)	7 (19)	7 (33)	
Not satisfied	7 (12)	3 (8)	4 (19)	
Virtual care ease of use				
Easy	43 (73)	27 (75)	14 (67)	.53
Neutral	13 (22)	8 (22)	5 (24)	
Difficult	3 (5)	1 (3)	2 (9)	
Appointment length during virtual visits compared to in-person visits				
Longer	7 (12)	3 (8.3)	3 (14)	.14
Same	17 (29)	14 (38.9)	3 (14)	
Shorter	35 (59)	19 (52.8)	15 (71)	

a Data suppressed for privacy.

### Usage/applications of virtual care

Provider experiences and use of virtual care were limited to telephone or video appointments. Prior to COVID-19, only 3% of providers engaged in frequent use of phone or video for scheduled appointments, while only occasionally use of phone (24%) or video (21%) was common. Most (87%) used phone occasionally or frequently for patient follow-up. During the pandemic, 78% reported using virtual care approaches for more than half of their appointments. In deciding whether to use phone or video for virtual appointments, the top 2 deciding factors were patient preference/comfort with the technology (53%), and the patient’s reason for visit (38%).

Providers reported low use of other virtual care modalities. Few providers regularly used email (27%), text messaging (19%), electronic assessments (5%), or electronic appointment requests (14%) and electronic appointment reminders (27%). Providers had limited interest in learning more about email (33%) and text messaging (24%). There was moderate interest in learning about electronic prescription renewal requests (40%) and appointment reminder (44%) functions in the future.

Providers indicated that the most appropriate appointments for virtual care included prescription refills, follow-up, and mental health appointments. Several providers (n = 13) noted that their virtual chronic disease-management appointments would be enhanced if patients had home-monitoring equipment (eg, blood pressure machines). More than 80% of respondents reported that virtual care was suitable for seniors without transportation, people living in very rural locations, and people with mobility challenges or disabilities.

### Provider satisfaction and experience with virtual care

Provider experience with virtual care was captured using appointment length, satisfaction, and ease of use. A majority (58%) indicated that their appointments were a bit shorter, much shorter, or lengths varied but it took them less time to get through the same number of appointments when using virtual care. Thirty-one percent reported that their appointments took about the same amount of time as in-person appointments, while 11% indicated that their appointments were longer, or lengths varied but it took them more time to get through the same number of appointments. Two-thirds of respondents (67%) were satisfied with their virtual care patient interactions; 11% expressed dissatisfaction. Appointment length, satisfaction, and ease of use scores were not significantly different between provider types (Table 2) or virtual care modality (Table 3). Satisfaction and ease of use scores were moderately positively correlated *r(57)* = 0.43, *P* = .003.

**Table 2.** Results for Mann-Whitney U test for satisfaction, ease of use, and appointment length.

	Mean (SD)		*P*
	Generalist (n = 36)	Specialist (n = 21)	
Satisfaction^a^	3.69 (1.01)	3.24 (1.30)	.14
Ease of use^b^	4.00 (0.79)	3.81 (0.93)	.49
Appointment length^c^	3.06 (1.29)	2.71 (1.49)	.23

a Likert scale 1 (less satisfied) to 5 (more satisfied).

b Likert scale 1 (less easy to use) to 5 (easier to use).

c Likert scale 1 (shorter) to 7 (longer).

**Table 3.** Results of the Kruskal-Wallis rank sum test for satisfaction, ease of use, and appointment length.

	Mean (SD)			*P*
	Phone (n = 32)	Phone and video (n = 24)	Zoom (n = 3)	
Satisfaction^a^	3.56 (1.05)	3.67 (1.17)	2.33 (1.15)	.08
Ease of use^b^	3.88 (0.79)	4.00 (0.89)	4.00 (1.00)	.85
Appointment length^c^	3.16 (1.53)	2.67 (1.09)	3.33 (2.31)	.55

a Likert scale 1 (less satisfied) to 5 (more satisfied).

b Likert scale 1 (less easy to use) to 5 (easier to use).

c Likert scale 1 (shorter) to 7 (longer).

#### Virtual care benefits

Providers indicated that the overwhelming benefits of virtual care versus in-person appointments were minimizing risk of COVID-19 exposure (97%) and reduced need for personal protective equipment (93%). Providers highlighted perceived benefits for their patients of not having to travel (89%) and appointments at times that were more convenient for them (66%). Forty-four percent of respondents reported that virtual care was more efficient for the provider, yet only 25% felt that the quality of care was better than or equal to in-person care. In open-ended responses, providers were positive about patient convenience, acknowledging that virtual care “*really helped patients as they don’t have to travel for an hour to see me*.” However, some were concerned about quality of care; as one specialist provider conveyed, “*I worry a great deal about missing something important because I can’t see their whole body. . .*”

Most respondents (83%) felt that video improved the appointment at least somewhat compared to phone. Providers highlighted 3 areas where video added value: (1) examinations, “*it allows for an abbreviated clinical examination*”; (2) non-verbal expressions, “*. . . many clues in observing the patient with video may not be picked up with the phone”*; and (3) building rapport, “*I get to actually SEE my patient. The phone is working most of the time, but I find it very impersonal. With video, I can connect much better with my patients.*” Yet, many providers acknowledged that while they used video, they predominantly used phone because of ease and technology issues, “*I rarely use video. Video is useful but cumbersome and slow*.” Despite technology issues, video-specific appointments were considered most useful when assessing skin conditions, mental health states, observing range of motion, and identifying the problem location.

#### Problems encountered with virtual care

Most problems encountered were specific to video appointments and centered on access to equipment or adequacy of connections. Administrative burdens were more varied and included additional workload for providers and staff, workflow challenges and new tasks (Table 4).

**Table 4.** Problems with technology and administration burdens encountered.

*Challenges with technology*	%
Patients not having access to required equipment	47
Patients’ internet services not fast or reliable	28
Provider unable to see or hear patient well	20
Provider’s internet services not fast or reliable	15
*Administrative burdens*	
Additional workload for administrative staff	30
Getting patients’ email addresses	24
Explaining to patients how videoconferencing works	24
Setting up the videoconference invite	21
Additional provider workload	22
Troubleshooting technology issues	17
Faxing forms/prescriptions that could normally be given to patient	17
Being unable to easily pass tasks off to staff when working from home	17

### Virtual care in the future

The vast majority (92%) of respondents indicated that they would do more virtual care visits in the future. Providers felt virtual visits would enhance their practice for (1) convenience, “*More convenient for patients—can just step away for a call from work or child care*”; (2) access “*Road travel in the winter can be challenging. Virtual care under these circumstances would be much better for patients*”; and (3) efficiency “*Prevent appointments that do not benefit at all from face-to-face visits . . . makes it easier following up on small issues that might not previously have justified a visit.*”

While providers supported virtual care use, they indicated virtual care could only continue if adequate remuneration for services continued to be provided, “*the fee codes are about right. They offer the patient the flexibility to choose what is right for them and us the financial ability to provide it . . . The old fee codes would not allow me to offer this*.” Most providers (73%) indicated that, prior to COVID-19, compensation did not incentivize virtual care. Given provincial remuneration changes due to the pandemic, most providers (78%) reported that the new compensation model incentivized virtual care. However, half the providers indicated that post-pandemic, they would use virtual care for less than 40% of their appointments.

## Discussion

The COVID-19 pandemic resulted in rapid uptake of virtual care solutions in both the US and Canada, requiring providers and patients to quickly adapt to new modalities of care delivery.^10,21,27^ Our participants similarly had a dramatic increase in virtual care use during the pandemic (from 3% pre-pandemic to 78% providing more than 60% of their visits virtually). While providers predominantly used telephone, they described benefits of video-supported appointments and expressed interest in expanding virtual care use beyond the virtual visit. Providers were generally satisfied with virtual care; however, they reported burdens and barriers to use similar to those found in studies of virtual healthcare implementation pre-pandemic.^16^

While most respondents felt that video improved the appointment at least somewhat over using the phone, satisfaction scores did not reflect this improvement. Provider satisfaction with Zoom, though not significant, trended lower than with other modalities. It is likely that provider reports of burdens and barriers, including connectivity issues, additional administrative work, and worries about security/privacy, contributed to lower satisfaction. A similar review of provider satisfaction with virtual care found that satisfaction was dependent on administrative support, compensation, and ease of use.^28^ As the pandemic forced providers to quickly adopt virtual care, many providers adopted platforms and strategies that were not well suited to their needs, lacked electronic medical record integration, and/or failed to identify other useful virtual care modalities. Similarly, limited uptake of wider virtual care applications was reported in Ontario, where only 9% of all virtual appointments in 2020 were video appointments.^10^ A unique finding of this study was the direct impact of poor internet access and bandwidth on providers, with over one quarter lacking the internet capacity necessary for video-supported appointments. This is an important issue in rural and mountainous regions of Canada where both practices and households lack suitable internet.^15^ Not surprisingly, the increase in virtual care has been more substantial in urban areas compared to rural.^2,14^

Our study found provider apprehension over missing important clinical indicators because of virtual care. This appears to be a concern among many providers, with 36% of 624 American primary care and specialty providers expressing worry about medical errors with use of virtual care technologies.^29^ While video appointments provide some visual cues, new and unfamiliar assessment styles can add a level of uncertainty to patient appointments for providers.^30^ The predominant use of telephone could compound this concern by removing all visual cues. However, there is little evidence to suggest that virtual care results in missed-diagnoses more than in-person.^31^ Additionally diagnostic accuracy appears to be comparable between in-person, video, and telephone visits.^31-33^

Despite provider reservations, there is substantial demand amongst patients for virtual care in a post-pandemic context. A pan-Canadian survey found 38% of 1800 patient respondents would choose phone, email, video, or text rather than in-person consultation as the first point-of-contact for needed healthcare in the future.^34^ The majority of providers (92%) wanted a continuation of virtual care visits in the future, however that was contingent on maintaining reimbursement rates. The concern for virtual care’s sustainability is whether funding will continue.^35^ In a statement from the British Columbia Ministry of Health and College of Physicians and Surgeons of B.C in September 2021, doctors were urged to resume in-person visits rather than virtual and that the funding adjustment made during the pandemic were “*temporary and could be cancelled.*”^36^ The loss of virtual care reimbursement would risk a step backwards for improving access to care, further disadvantaging the patients who benefited most from virtual care. Importantly, virtual care should not be seen as a substitute for in-person care, but an opportunity to improve and expand how we deliver patient-centered care.^37^

### Implications

To promote continued and optimized virtual care provision, several avenues can be pursued. Practice-level changes to align with perceptions of suitability for patients include investing in high-speed internet where available; taking advantage of virtual platforms provided freely through their Health Authorities; training care team members and exploring virtual care options such as email, text messaging and adding a patient portal; and optimizing office workflow through reassigning tasks (Table 4). Research is needed to explore patient preferences and continue to identify best practices, barriers, patient safety, and facilitators to enhance knowledge translation through local provider groups. System-wide changes, perhaps more aspirational, include provincial and federal funding to both improve connectivity and provide educational, physical, and change-support resources for patients and providers in rural communities.^38-40^ Integration of virtual care into practice (eg, workflow, patient care coordination, usability) is a complex process.^41^ Therefore, virtual care integration and optimization will require a systems-level perspective on such complexities to ensure that funding allocations address systemic barriers to long-term adoption.

### Limitations/future research

The study sample may not have been represented of rural primary care providers due to small sample size and self-selection bias. However, the survey response was comparable to previously deployed survey studies.^42^ Additionally, provider responses were collected after the first wave of the pandemic in BC, which may not accurately reflect providers’ virtual care experiences over time. A follow-up study of providers from these rural practices, including an in-depth examination of their virtual care transitions, would provide valuable comparative data showing changes in usage patterns over time.

The sampled providers mostly used telephone visits, however our questions on virtual care experiences and satisfaction grouped telephone and video together as “virtual care.” Therefore, specific experiences with telephone versus video use may have been missed. Future work should examine use and barriers to both synchronous and asynchronous approaches to reflect the entirety of virtual care usage. Although providers spoke about their perceptions of patient concerns with virtual care, these may not be reflective of patients actual concerns and could be validated in a future study.

## Conclusions

Use of virtual care resulted in high rural generalist and specialist provider satisfaction and shorter visits for most while not causing undue increase in workload. A unique finding was the limited use of video technology due in part to poor internet access and bandwidth for providers, more problematic in rural and remote communities. Lack of digital infrastructure and resources prevent widespread adoption in rural communities, for both providers and patients. Despite challenges, our results showed provider willingness to engage with virtual care, which provides an opportunity to integrate these services long-term. People residing in remote communities are disadvantaged in terms of health and healthcare, highlighting the importance of finding novel solutions to address inequalities in access through practice changes; appropriate funding for virtual care, and additional federal, provincial, and local support for high-speed Internet access and education to enhance digital literacy. Without proactive solutions to address these inequities, rural providers will be unable to capitalize on the full potential that virtual care offers and care quality may suffer, unable to keep pace with the rapidly evolving virtual care landscape.

## Supplemental Material

sj-pdf-1-his-10.1177_11786329221096033 – Supplemental material for Has Virtual Care Arrived? A Survey of Rural Canadian Providers During the Early Stages of the COVID-19 Pandemic Click here for additional data file. Supplemental material, sj-pdf-1-his-10.1177_11786329221096033 for Has Virtual Care Arrived? A Survey of Rural Canadian Providers During the Early Stages of the COVID-19 Pandemic by Lindsay Burton, Kathy L Rush, Mindy A Smith, Matthias Görges, Leanne M Currie, Selena Davis, Mona Mattei and Jennifer Ellis in Health Services Insights
