# Supplementary material for: Has Virtual Care Arrived? A Survey of Rural Canadian Providers During the Early Stages of the COVID-19 Pandemic
Source: Health Serv Insights. 2022 May 17;15:11786329221096033. doi: 10.1177/11786329221096033 (PMC9118397; doi:10.1177/11786329221096033)
Supplement: sj-pdf-1-his-10.1177_11786329221096033 – Supplemental material for Has Virtual Care Arrived? A Survey of Rural Canadian Providers During the Early Stages of the COVID-19 Pandemic [file sj-pdf-1-his-10.1177_11786329221096033.pdf]

Divisions of Family Practice
A GPSC initiative

Platform

1. What systems/platforms do you use most of the time to conduct virtual health appointments (select all that apply)?

- [ ] Phone
- [ ] Doxy.me
- [ ] Zoom
- [ ] Input Health
- [ ] Other (please specify):

2. Do you use both phone and video platforms, depending on the appointment?

- ( ) Yes
- ( ) No, I use only phone
- ( ) No, I use only video

3. How do you determine whether to utilize phone or video for a particular appointment?

4. Does having video improve the appointment, compared to using the phone?

- ( ) Very much so
- ( ) Somewhat
- ( ) Not at all

5. What is the value add of having video?

6. What are some of the reasons you chose the system/platform you are using (select all that apply)?

- [ ] Convenience
- [ ] Familiar with the technology
- [ ] Ease of use
- [ ] Lower cost
- [ ] Felt patients would be familiar/comfortable with the technology
- [ ] Directed to use the platform by Interior Health
- [ ] Wanted to be able to see patients as well as hear them in appointments
- [ ] Don't feel you will be using virtual health long term so did not want to invest in a system

7. How would you describe your use of virtual health prior to the COVID-19 pandemic (select all that apply)?

- [ ] Never used any form of virtual health
- [ ] Occasional use of phone to follow up with patients
- [ ] Frequent use of phone to follow up with patients
- [ ] Occasional use of phone for appointments with patients
- [ ] Frequent use of phone for appointments with patients
- [ ] Occasional use of video for appointments with patients
- [ ] Frequent use of video for appointments with patients

8. Approximately how many appointments are you doing via virtual health per day currently?

- ○ 1 to 5
- ○ 6 to 10
- ○ 11 to 15
- ○ 16 to 20
- ○ 21 to 25
- ○ 26 or more

9. Approximately how many in-person appointments were you doing per day prior to the COVID-19 pandemic?

- ◯ 1 to 5
- ◯ 16 to 20
- ◯ 6 to 10
- ◯ 21 to 25
- ◯ 11 to 15
- ◯ 26 or more

10. Approximately what percentage of appointments are you conduct via virtual health currently?

- ◯ Less than 5%
- ◯ 31 to 40%
- ◯ 5 to 10%
- ◯ 41 to 60%
- ◯ 11 to 20%
- ◯ 61 to 80%
- ◯ 21 to 30%
- ◯ More than 81%

11. Approximately what percentage of appointments did you conduct via virtual health prior to the COVID-19 pandemic?

- ◯ Less than 5%
- ◯ 31 to 40%
- ◯ 5 to 10%
- ◯ 41 to 60%
- ◯ 11 to 20%
- ◯ 61 to 80%
- ◯ 21 to 30%
- ◯ More than 81%

12. Is the length of your appointments longer or shorter when providing care virtually?

- ◯ Much longer
- ◯ A bit longer
- ◯ About the same
- ◯ A bit shorter
- ◯ Much shorter
- ◯ It varies from appointment to appointment but overall it takes me less time to get through the same number of appointments than in-person
- ◯ It varies from appointment to appointment but overall it takes me more time to get through the same number of appointments than in-person

13. Do you routinely utilize any other virtual health solutions to connect with patients (before or during the COVID-19 pandemic) (select all that apply)?

- ☐ Email advice or check-ins

- [ ] Text messaging advice or check-ins
- [ ] Online assessments
- [ ] Clinic patient portal to access their health record
- [ ] Electronic prescription renewal request
- [ ] Electronic appointment booking requests
- [ ] Electronic appointment reminders

14. What other virtual health solutions to connect with patients are you interested in learning more about (select all that apply)?

- [ ] Email advice or check-ins
- [ ] Text messaging advice or check-ins
- [ ] Online assessments
- [ ] Clinic patient portal to access their health record
- [ ] Electronic prescription renewal request
- [ ] Electronic appointment booking requests
- [ ] Electronic appointment reminders

Divisions of Family Practice
A GPSC initiative

Experience and Satisfaction

The questions in this section apply to whatever form of virtual health you are using with patients (phone or video).

15. How satisfied are you with your interaction with patients and the care you are providing when providing care virtually?

- Very satisfied
- Satisfied
- Neutral
- Not very satisfied
- Not satisfied at all

16. How has providing care virtually changed your experience of practicing medicine (e.g. has it improved your satisfaction, decreased your satisfaction, improved your patient interactions)?

17. Overall, how easy or difficult is it for you to conduct visits virtually?

- Very easy
- Easy
- Neither easy nor difficult
- Difficult
- Very difficult

18. What, if any, problems or concerns have you encountered during your virtual health visits (select all that apply)?

- [ ] I do not have any problems or concerns
- [ ] I often have equipment problems such as computer glitches or battery problems
- [ ] I do not have access to the required equipment
- [ ] My internet services are not fast or reliable enough
- [ ] My cell phone service is not reliable enough
- [ ] I am not familiar or comfortable with using the technology
- [ ] It creates additional workload for me
- [ ] It creates additional workload for my administrative staff
- [ ] I don’t have anyone to call if I have a technology problem
- [ ] I am concerned of the privacy of the visit
- [ ] Patients are concerned about the privacy of the visit
- [ ] I often cannot not hear or see the patient well enough
- [ ] I do not have an appropriate space to do virtual health appointments in my home
- [ ] I do not have an appropriate space to do virtual health appointments in my clinic
- [ ] Patients do not have access to the required equipment
- [ ] Patients internet services are not fast or reliable enough
- [ ] I have not had good enough support for practice change
- [ ] Other (please specify):

19. If you indicated that doing virtual health visits creates too much additional workload for you or your administrative staff, can you describe this additional workload?

20. What types of appointments are most suitable for virtual appointment?

For example, first appointments, follow-up appointments, preventative care, prescription refills, counselling, chronic disease management etc.

21. Overall, how easy or difficult is it for your patients to participate in virtual health visits?

- Very easy
- Easy
- Neither easy nor difficult
- Difficult
- Very difficult

22. Are there subsets of patients foregoing care because they cannot come in for normal care?

- Yes
- No
- Not sure

23. What subsets of patients are foregoing care?

24. Are there subsets of patients seeking care more frequently because virtual health is an option?

- Yes
- No
- Not sure

25. What subsets of patients are seeking care more frequently?

26. What vulnerable populations might be served well by virtual health (please select all that apply)?

- [ ] Homeless people
- [ ] Seniors with no transportation
- [ ] People living in very rural locations
- [ ] People with mobility challenges or disabilities
- [ ] Other (please specify):

27. Are any of the following costs a barrier to you doing virtual health appointments by video (please select all that apply)?

- [ ] Equipment costs
- [ ] Software subscription/licensing costs
- [ ] System maintenance costs
- [ ] Other (please specify):

28. What are the benefits of doing virtual health appointments compared to in-person appointments (please select all that apply)?

- [ ] Minimizes risk of exposure to COVID-19 for providers and patients
- [ ] Minimizes use of PPE
- [ ] Easier for patients to get to appointment (i.e. they do not have to travel to clinic)
- [ ] Can be undertaken at times more convenient to patients
- [ ] Quality of visit is equal to in person
- [ ] Quality of visit is better than in person
- [ ] More opportunity to focus on preventative care
- [ ] It is more efficient for the provider
- [ ] Other (please specify):

Divisions of Family Practice
A GPSC initiative

Use of Virtual Health in the Future

29. Do you think you will undertake virtual health visits more than you did in the past when the COVID-19 pandemic is over?

- ○ Yes
- ○ No

30. Approximately what percentage of appointments are you conduct via virtual health currently?

○ Less than 5%	○ 31 to 40%
○ 5 to 10%	○ 41 to 60%
○ 11 to 20%	○ 61 to 80%
○ 21 to 30%	○ More than 81%

31. How might virtual health enhance or complement the services that you provide in the future?

32. Do the fee code rules (as they were before the COVID-19 pandemic) support and incentivize the use of virtual health?

- ○ Yes
- ○ No

33. Do the fee code rules implemented for the COVID-19 pandemic support and incentivize the use of virtual health?

- ○ Yes
- ○ No

34. How could the fee codes and fee code rules be changed to better incentivize and support virtual health?

35. Are you interested in further supports to expand or enhance your use of virtual health now or in the future (e.g. extra training, troubleshooting support) (select all that apply)?

- [ ] Yes, I am interested in additional supports now
- [ ] Yes, I am interested in additional supports after the COVID-19 pandemic is over
- [ ] No

36. What supports would be helpful to you?

37. Do you need support in addressing privacy and consent concerns associated with virtual health?

- ○ Yes, I am interested in additional supports now
- ○ Yes, I am interested in additional supports after the COVID-19 pandemic is over
- ○ No

Patient Panel Size and Virtual Health

38. Do you feel the efficiencies associated with engaging in virtual health would enable you to add more patients to your panel?

- Seeing patients virtually is more efficient, but I am not interested in attaching more patients because the efficiencies of virtual care are enabling me to have better work/life balance
- Yes, the efficiencies of virtual care would allow me to manage a larger patient panel, and I will consider attaching more patients
- Once the COVID-19 pandemic is over, I don’t expect to be doing enough virtual care to enable me to attach more patients
- No, virtual care is not more efficient and would not enable me to attach more patients

Use of other Platforms by Patients

39. Are any of your patients using online services such as Babylon, Maple or other platforms that you are aware of?

- Yes
- No

40. Please list which ones you are aware of them using:

41. Why do you think they are using these other platforms and how has patient use of these other platforms impacted their care?

Demographics

42. What gender do you identify with?

- Male
- Female
- Non-binary
- Other

43. What is your age?

- 25 to 34
- 35 to 44
- 45 to 54
- 55 to 64
- 65 or older

44. Are you a:

- General practitioner in family practice
- Nurse practitioner in family practice
- General practitioner or nurse practitioner in a specialized practice
- Hospitalist
- Specialist practitioner
- Other (please specify):

Thank you for taking the survey.
